# Targeting Novel Regulated Cell Death: Pyroptosis, Necroptosis, and Ferroptosis in Diabetic Retinopathy

**DOI:** 10.3389/fcell.2022.932886

**Published:** 2022-06-23

**Authors:** Sheng Gao, Yun Zhang, Meixia Zhang

**Affiliations:** ^1^ Department of Ophthalmology, West China Hospital, Sichuan University, Chengdu, China; ^2^ Research Laboratory of Macular Disease, West China Hospital, Sichuan University, Chengdu, China

**Keywords:** diabetic retinopathy, regulated cell death, pyroptosis, necroptosis, ferroptosis

## Abstract

Diabetic retinopathy (DR) is one of the primary causes of visual impairment in the working-age population. Retinal cell death is recognized as a prominent feature in the pathological changes of DR. Several types of cell death occurrence have been confirmed in DR, which might be the underlying mechanisms of retinal cell loss. Regulated cell death (RCD) originates from too intense or prolonged perturbations of the intracellular or extracellular microenvironment for adaptative responses to cope with stress and restore cellular homeostasis. Pyroptosis, necroptosis, and ferroptosis represent the novel discovered RCD forms, which contribute to retinal cell death in the pathogenesis of DR. This evidence provides new therapeutic targets for DR. In this review, we summarize the mechanisms of three types of RCD and analyse recent advances on the association between novel RCD and DR, aiming to provide new insights into the underlying pathogenic mechanisms and propose a potential new strategy for DR therapy.

## Introduction

Diabetes mellitus is a global epidemic and leads to various complications, including macrovascular and microvascular complications, such as cardiovascular disease, stroke, and kidney disease (Shaw et al., 2010). Diabetic retinopathy (DR) is one of the microvascular complications of diabetes mellitus and is recognized as the primary cause of visual impairment in the working-age population (Antonetti et al., 2012). The Wisconsin Epidemiologic Study of Diabetic Retinopathy (WESDR) study reported that approximately 75% of diabetes patients would develop DR 10 years after diabetes diagnosis (Klein et al., 1984a; Klein et al., 1984b). For DR patients at baseline, approximately 67% of patients would develop to the severe stage of DR, and approximately 20% of patients developed proliferative DR (PDR) or diabetic macular edema (DME) (Klein et al., 1984c; Klein et al., 1995). The main vascular pathologic changes of DR caused by chronic hyperglycemia include endothelial cell death, thickening of the capillary membrane, increased vascular permeability, retinal tissue ischemia and capillary leakage, and the release of various vasoactive substances, leading to neovascularization (Wong et al., 2016). Clinically, DR can be classified into nonproliferative DR (NPDR) and PDR according to whether retinal vascularization occurs. In the early stage, NPDR is characterized by microaneurysms, increasing vascular permeability, retinal edema and hemorrhage, and vascular occlusion, which can be subdivided further into mild, moderate and severe NPDR. During severe NPDR, vascular occlusion is aggravated, and various growth factors are secreted, signaling to the retina to form new vessels to provide blood and oxygen (Cunha-Vaz et al., 2014; Daruich et al., 2018). Subsequently, PDR develops in some patients and is characterized by the formation of new blood vessels in the retinal vasculature. Due to the instability and dysfunction of new blood vessels, unsealed neovascular vessels leak erythrocytes and plasma into surrounding retinal tissue, resulting in vitreous hemorrhage and retinal detachment, which severely impair visual acuity (Simó et al., 2006).

Most studies of DR to date have focused on retinal microangiopathy, which has been directly implicated in visual impairments (Wong et al., 2016). The roles of retinal neurodegeneration and inflammation in the development of DR are being studied (Forrester et al., 2020). Pathological changes in the retina have been detected, and all retinal cell types are affected by diabetes. Histologically, retinal neurons, glia, endothelial cells and pericytes in the retina are cohesive structures and link with retinal pigment epithelium (RPE) cells to constitute retinal tissue (Meng et al., 2021). Indeed, loss of these retinal cells seems to be a prominent pathologic change and is related to the pathogenesis of DR. Increasing evidence has demonstrated retinal cell death occurrence in DR. However, identifying potential modes of cell death is complex, and the death pathway and markers are still being explored.

According to the guidelines for the definition and interpretation of cell death formulated by the Nomenclature Committee on Cell Death 2018, regulated cell death (RCD) is defined as the form of cell death that results from the activation of signal transduction modules and hence can be pharmacologically or genetically modulated (Galluzzi et al., 2018). It is involved in two completely opposed scenarios. One is RCD occurrence as an effector of physiological process for development or tissue regeneration without any exogenous environmental perturbation. The other is RCD occurrence originating from intracellular or extracellular microenvironmental perturbations when such perturbations are too prolonged or intense for adaptative responses to deal with stress and restore cellular homeostasis (Galluzzi et al., 2018). Apoptosis, as a type of RCD, is the most studied cell death mode in DR early and has well-defined features (Kang and Yang, 2020). Recent studies have revealed several novel RCDs involved in the pathogenesis of DR, including pyroptosis, necroptosis and ferroptosis. PANoptosis and cuproptosis are novel RCD pathways recently discovered and named. PANoptosis is a unique inflammatory RCD with characteristic of apoptosis, necroptosis and pyroptosis that cannot be explained by any of these three RCD pathway alone (Wang and Kanneganti, 2021). Cuproptosis is a copper-triggered and lipoylated tricarboxylic acid cycle proteins-mediated modality of mitochondrial cell death (Tsvetkov et al., 2022). These two RCD are lacking in direct evidence of the association with DR currently. In this review, we focus on the three types of novel RCD, including pyroptosis, necroptosis and ferroptosis, and summarize recent advances in the association between cell death and DR. Finally, we propose that novel RCD is a potential therapeutic target for DR treatment in the future.

## Novel Regulated Cell Death in Diabetic Retinopathy

### Overview of Pyroptosis

**FIGURE 1:**
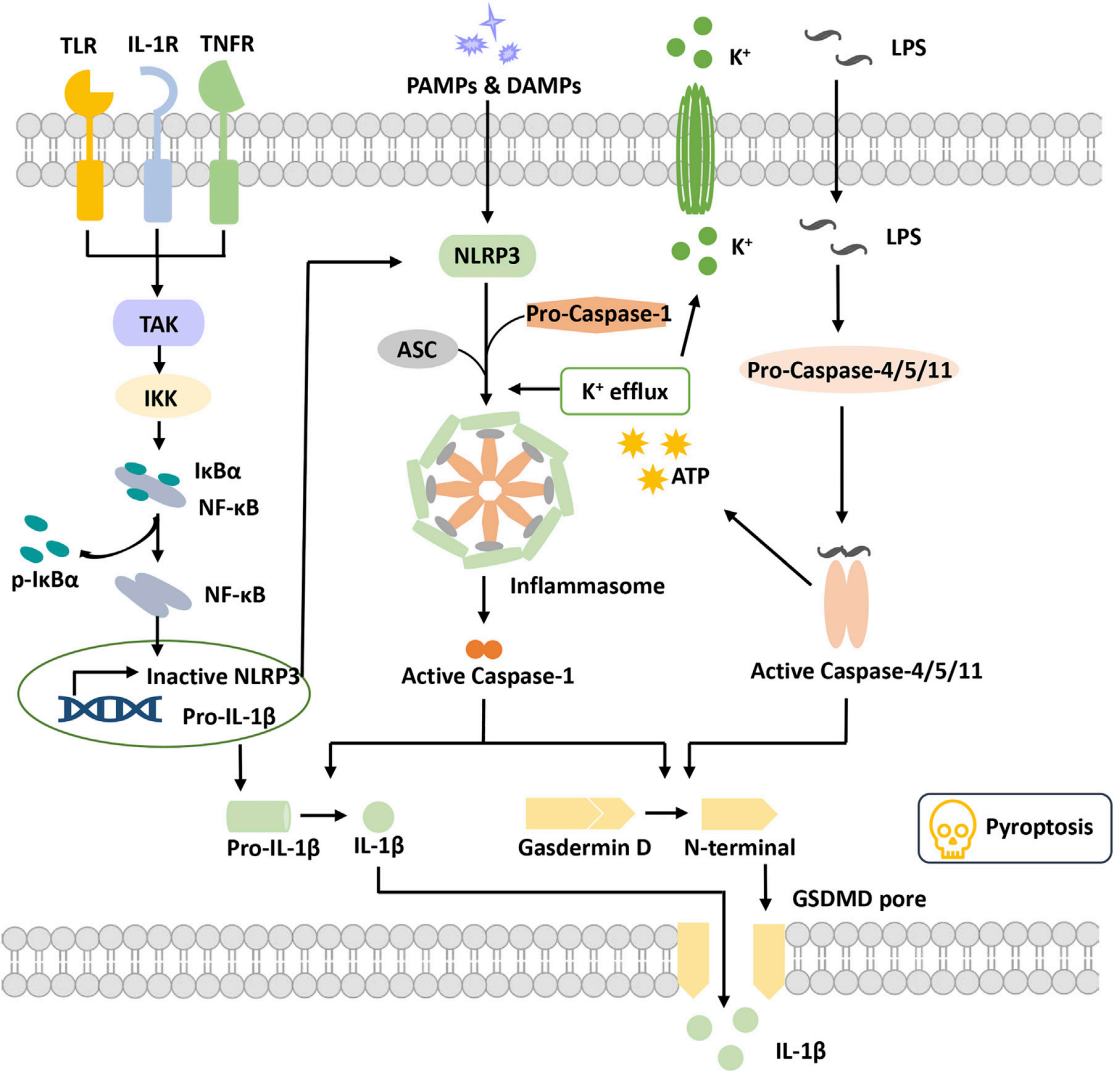
Overview of pyroptosis. Pyroptotic priming signals are usually triggered by endogenous cytokines and activate the NF-κB signaling pathway to induce the transcription of inactive NLRP3 and pro-IL-1β. Activation signals are triggered by the stimulation of DAMPs or PAMPs, stimulating NLRP3 oligomerization to recruit ASC and pro-caspase-1 and then form the NLRP3 inflammasome. Activated caspase-1 cleaves GSDMD to release the N-terminal domain and form the plasma membrane pore, leading to pyroptosis. Activated caspase-1 promotes the maturation and secretion of IL-1β. In addition, LPS binds to pro-caspase-4/5/11 to activate it, inducing pyroptosis via cleavage of GSDMD and formation of pores. Activated caspase-4/5/11 can also induce K+ efflux and activate the NLRP3 inflammasome. Abbreviations: ASC, apoptosis speck-like protein; DAMPs, damage-associated molecular patterns; GSDMD, gasdermin D; IKKα/β, IkB kinase α/β; IL-1β, interleukin-1β; LPS, lipopolysaccharide; PAMPs, pathogen-associated molecular patterns; TAK1, beta-activated kinase 1; TLR, toll-like receptor.

Pyroptosis is a pathway of RCD that depends on the pore-forming activity of the gasdermin protein family with an inflammatory response. As an important part of the body’s innate immune response, it plays an indispensable role in antagonizing pathogen infection and sensing endogenous danger signals (Man et al., 2017; Shi et al., 2017) (Figure 1). Cells are stimulated by various stimuli to form a multiprotein complex called the inflammasome, which can activate the cysteine-containing aspartate-specific protease Caspase-1. Activated caspase-1 cleaves gasdermin D (GSDMD) and releases its N-terminal domain to punch in the cell membrane and form membrane pores, causing cell swelling and rupture and pyroptosis (Ding et al., 2016; Liu et al., 2016). Meanwhile, activated caspase-1 matures inflammatory factors (such as interleukin (IL)-1, IL-18, etc.) that are released to the extracellular matrix through ruptured cell membranes. Mature IL-1 acts as a potent proinflammatory mediator to recruit innate immune cells to infection sites and regulate adaptive immune cells, while mature IL-18 promotes the production of interferon-γ (IFN-γ) and enhances the cytolytic activity of natural killer T cells and T cells, contributing to the elimination of pathogenic microbial infection or abnormal cells *in vivo* (Bergsbaken et al., 2009). Pyroptosis-mediated cell death is associated with the progression of various human diseases, including infectious diseases, metabolic diseases, autoimmune diseases, neurological-related diseases, cardiovascular diseases, and ocular diseases (Man et al., 2017; Van Gorp et al., 2019; Zeng et al., 2019; McKenzie et al., 2020; Sharma and Kanneganti, 2021). Recently, a growing amount of evidence reports that inflammasome activation plays a significant role in triggering pyroptosis-mediated cell death and promotes DR progression (Gu et al., 2019).

The inflammasome was first proposed by Professor J. Tschopp in 2002 (Martinon et al., 2002). It is a complex constructed by pattern recognition receptors (PRRs), apoptosis speck-like protein (ASC) and pro-caspase-1 when cells are suffering danger signal stimulations. It has the function of activating caspase-1 and is also a core of the occurrence of pyroptosis.

Pattern recognition receptors (PRRs) are a class of immune receptors mainly expressed in immune cells that recognize multiple damage-related molecular patterns (DAMPs) or pathogen-related molecular patterns (PAMPs) of invading microorganisms (Dostert et al., 2008). They are the first process of the innate immune system to resist infection and include the NOD-like receptor (NLR), AIM2-like receptor (ALR) families, C-type (carbohydrate-binding lectin domain) lectin receptor (CLR), Toll-like receptor (TLR), and retinoic acid–inducible gene (RIG)-I–like receptor (RLR), (Brubaker et al., 2015). Among them, multiple PRRs of the NLR family and the ALR family have been confirmed to construct inflammasomes and mediate pyroptosis occurrence. The NLRP3 (NOD-, LRR-, PYD containing 3) inflammasome is currently the most studied inflammasome. NLRP3 is composed of three domains, pyrin domain (PYD), nucleotide-binding and oligomerization domain (NACHT) and leucine-rich repeat (LRR), which cannot directly recruit and activate caspase-1 and requires the participation of the adaptor protein ASC(Swanson et al., 2019). Canonical NLRP3 inflammasome activation requires two signals. The first priming signal, triggered by endogenous factors or microorganisms, causes the upregulation of NLRP3 and pro-IL-1 expression through activating the NF-κB signaling pathway (Bauernfeind et al., 2009). The second activation signal is triggered by various stimuli and further induces K^+^ efflux, Cl-efflux, etc. These events promote NLRP3 oligomerization to recruit both ASC and pro-caspase-1 and to form an activated NLRP3 inflammasome (Gritsenko et al., 2020). Activated caspase-1 cleaves gasdermin D to release the N-terminal domain and induce the occurrence of pyroptosis. Meanwhile, caspase-1 cleaves pro-IL-1β into IL-1β and releases it into the extracellular matrix (He et al., 2016). Nonclassical NLRP3 inflammasome activation is induced by lipopolysaccharide (LPS) and activates caspase-4/5/11, which in turn triggers pannexin-1 channels to induce K^+^ efflux and adenosine triphosphate (ATP) release (Rühl and Broz, 2015). Subsequently, the NLRP3 inflammasome is activated to release mature IL-1. Activated caspase-4/5/11 also induced pyroptosis by cleaving gasdermin D and forming pores on the cell membrane (Kayagaki et al., 2011; Hagar et al., 2013).

ASC, an apoptosis speck-like protein containing a caspase-recruitment domain (CARD) and PYD as an adaptor protein, is part of various inflammasome complexes. Through the PYD-PYD and CARD-CARD-mediated stacking function, ASC can anchor and activate more caspase-1 and enhance the cell response to stimulation signals to achieve signal amplification (Dick et al., 2016; Agrawal and Jha, 2020). For PRRs with PYD domains, such as NLRP3 and absent in melanoma 2 (AIM2), inflammasome formation is strictly dependent on the ASC protein, and ASC deficiency completely blocks the assembly and activation of these classes of inflammasomes (Sharma and De Alba, 2021).

Caspase family proteins are a class of cysteine-dependent endoproteases that specifically hydrolyze their substrates after specific aspartic acid residues. Their function is involved in the regulation of apoptosis, pyroptosis and necroptosis (Van Opdenbosch and Lamkanfi, 2019). Caspases are usually present in an inactive zymogenic state, and when stimulated by upstream signals, caspases present dimerization-induced conformational changes to undergo autoactivation and exert a proteolytic enzyme function (Lamkanfi et al., 2002). At present, 11 kinds of caspase proteins have been identified in humans, among which caspase-1, 4, 5, and 11 are involved in pyroptosis and caspase-4, 5, and 11 participate in the NLRP3 nonclassical activation pathway (Kayagaki et al., 2015; Schmid-Burgk et al., 2015; Shi et al., 2015). Caspase-1 is a component of multiple inflammasomes and is activated depending on the inflammasome complexes to form a fully functional protease (p20/p10). Activated Caspase enzymatically digested its substrates (GSDMD, pro-IL-1 and pro-IL-18), causing pyroptosis and the release of inflammatory factors (Shi et al., 2015).

Gasdermin (GSDM) protein is an effector protein. GSDMA-E incorporates two domains, a C-terminal suppressive domain and an N-terminal activator domain (Liu et al., 2021). Normally, the two domains of the GSDMs interact to keep the GSDMs in an autoinhibited state. When the C-terminal suppressive domain is hydrolyzed, the N-terminal activator domain will bind with specific lipid components, such as phosphoinositide and cardiolipin, in the cell membrane, penetrating holes and destroying the cell membrane integrity (Ding et al., 2016). GSDMD is the first protein of the gasdermin family found to be involved in pyroptosis, which can be directly activated by activated caspase-1/4/5/8/11 (He et al., 2015; Shi et al., 2015; Liu et al., 2016). The holes formed by GSDMD are approximately 22 nm in diameter and have negative electricity in the pore, which is very important to release mature IL-1β (positive electricity on the surface) (Xia et al., 2021). In addition to GSDMD, activation of other gasdermin family members also causes pyroptosis, but these are independent of the inflammasome and without inflammatory cytokine release (Wang et al., 2017). Thus, the potential relationship between pyroptosis and DR progression remains to be investigated. Numerous studies are ongoing to explore the molecular mechanism of pyroptosis and its potential role in DR pathogenesis.

### Pyroptosis and Diabetic Retinopathy

It was recently found that pyroptosis may occur in multiple types of retinal cells in DR models *in vitro* and *in vivo*, including endothelial cells, pericytes, retinal neurons, Müller cells, microglia and retinal pigmental epithelium (RPE) cells. Histologically, retinal neurons, glia cells (Müller cells, astrocytes, and microglia), and blood cells (endothelial cells and pericytes) in the retina compose an important structure named the retinal neurovascular unit (Hammes, 2018).

Vascular endothelial cells and pericytes are important components of the retinal microvasculature and internal blood–retina barrier, whose normal function and complete structure rely on interactions of endothelial cells and pericytes (Huang, 2020). NLRP3/caspase-1 activation and IL-1β release have been reported in retinal endothelial cells (RECs) and retinal microvascular endothelial cells (RMECs) in multiple *in vitro* and *in vivo* models of DR (Chen et al., 2017; Jiang et al., 2017; Gu et al., 2019). The levels of and cytosolic high-mobility group box 1 (HMGB1) are significantly increased in high glucose-induced RECs, which has been reported to activate HMGB1 and are associated with the NLRP3 inflammasome (Berger et al., 2016; Jiang et al., 2017). Meanwhile, cleaved caspase-1 and activated IL-1β were markedly increased in RECs incubated in high glucose compared with controls. Epac1 (a downstream mediator of β-adrenergic receptors) agonist and/or NLRP3 siRNA can effectively reduce hyperglycemia-induced increases in TLR4, HMGB1, cleaved caspase-1, and IL-1β in RECs(Jiang et al., 2017). Another study showed that hyperglycemia significantly increased the expression of NLRP3, ASC, and proinflammatory cytokines in high glucose-induced RMECs and diabetic rat retinas. Upregulation of mature IL-1β, IL-18 secretion and caspase-1 cleavage was also observed compared with controls. NLRP3 silencing or thioredoxin interacting protein (TXNIP) silencing blocked these alterations in hyperglycemia-induced RMEC and diabetic rat retinas, indicating that the TXNIP pathway mediates NLRP3 inflammasome activation and that inflammasome activation leads to inflammation in DR (Chen et al., 2017). Gu et al. found that the activity and expression of caspase-1 and IL-1β were markedly increased in HG-induced HRMEC and clinical vitreous samples, suggesting that pyroptosis is involved in the pathogenesis of DR. In addition, downregulated miR-590–3p and upregulated NLRP1/NOX4 levels were observed in RMECs of DR, which means downregulationg of miR-590–3p promoted pyroptosis by targeting NLRP1 and activating the NOX4-mediated pathway (Gu et al., 2019). These results suggest that canonical pyroptosis pathway may occur in RECs and RMECs, and targeting NLRP3 and caspase-1 may be a therapeutic strategy to inhibit pyroptosis in DR.

Pericyte coverage can provide structural support to the vessel wall and regulate the expression of tight junction proteins in the surrounding endothelial cells (Lechner et al., 2017). A recent study showed that high glucose induces NLRP3-caspase-1-GSDMD activation and pore formation in a dose- and time-dependent manner and results in the release of the inflammatory cytokines IL-1β and IL-18 and lactate dehydrogenase (LDH) from human retinal pericytes, which are all signs of retinal pericyte pyroptosis (Gan et al., 2020). Caspase-3 or caspase-1 inhibitors or GSDMD silencing synergistical treatment blunts these effects in part by reversing high glucose-induced inflammation and pyroptosis (Gan et al., 2020). In addition, advanced glycation end products (AGEs) play an important role in the depletion of pericytes by activating intracellular oxidative stress and inflammatory responses through binding to receptors for AGEs on the cell surface (Maeda et al., 2014). Another study used AGE-modified bovine serum albumin to simulate the DR environment and found that it induced activated caspase-1 and gasdermin D cleavage, increased the release of IL-1β, IL-18 and LDH, and reduced cell viability, indicating the occurrence of caspase-1-mediated pyroptosis in retinal pericytes (Yu et al., 2021). In this process, long noncoding RNA myocardial infarction-associated transcripts relieved the depression of caspase-1 by sponging miR-342–3p, thus facilitating caspase-1-dependent pericyte pyroptosis, which may provide novel insight into pericyte loss mechanisms and DR treatment (Yu et al., 2021). These studies suggest that pyroptotic pericyte loss may occur during DR development and blocking GSDMD and caspase-1 may maintain pericyte viability potentially.

Retinal neurons are the major cells that transmit light signals and form vision, wherein retinal ganglion cells (RGCs) are the output neurons integrating information (Völgyi, 2020). Multiple studies have confirmed retail neuronal alterations in early DR, such as neuronal degeneration and neuronal death, even earlier than clinical vasculopathy. Retinal ganglion cells undergo inflammatory caspase-mediated pyroptosis (Thomas et al., 2017). In the diabetic rat model, NLRP3, ASC, and caspase-1 were specifically located in the ganglion cell layer and the inner and outer nuclear layers, as shown by immunohistochemistry. The number of cells expressing NLRP3, ASC, and caspase-1 was markedly increased in the diabetic rat group. The suppression of NLRP3 inflammasome activation inhibits the expression of IL-1β and IL-18 (Yin et al., 2017). Pyroptosis mediated by inflammatory caspases can occur in RGCs in other retinal diseases. In the partial optic nerve crush injury model, NLRP3 was upregulated in retinal microglial cells and subsequently led to the upregulation of caspase-1 and IL-1β. Knocking out the NLRP3 gene delays RGC loss after partial optic nerve crush injury (Puyang et al., 2016). Pyroptosis is involved in retinal ischemic damage and promotes RGC death via the caspase-8-HIF-1α-NLRP12/NLRP3/NLRP4 pathway in acute glaucoma (Chen H. et al., 2020). Pyroptosis also participates in photoreceptor degeneration after retinal detachment (Li et al., 2020). RGC death *via* caspase-dependent mechanisms primarily occurs in ocular injury and progressive degenerative diseases of the eye, and future studies need to determine whether all retinal neurons undergo pyroptotic cell death in DR.

Müller cells, as the most abundant and widely distributed macroglia in the retina, participate in structural support and metabolic nutrition in a healthy retina (Coughlin et al., 2017). Considering that DR is viewed as a potential chronic inflammatory disease, Müller cells have long been speculated to respond to high glucose levels by undergoing pyroptotic cell death rather than apoptosis (Feenstra et al., 2013). Early studies showed that Müller cell death occurred in diabetic retinopathy and that dying Müller cells presented hypertrophy consistent with the sign that during pyroptosis, cells swell rather than shrink, as observed in apoptotic cell death (Hori and Mukai, 1980; Mizutani et al., 1998). Clear identification of retinal cells dying *via* pyroptosis was difficult given the lack of studies of specific proteins associated with pyroptosis execution at that time. Subsequent studies have confirmed that elevated glucose contributes to the activation of caspase-1 and IL-1β production in a cultured rat Müller cell model (Trueblood et al., 2011). Using minocycline inhibits caspase-1 activity and the production of IL-1β and subsequent cell death in retinal Müller cells of diabetic mice (Vincent and Mohr, 2007). These studies demonstrated Müller cell loss in DR and suggested that cell death might occur *via* a pyroptotic mechanism. A study published in 2020 found that the reactive oxygen species (ROS)/TXNIP/NLRP3 inflammasome axis was activated in high glucose-stimulated Müller cells *in vitro* and in Müller cells of DR mice. The levels of NLRP3 inflammasome-associated proteins, including ASC, cleaved caspase-1, and cleaved IL-1β, were increased by high glucose treatment of Müller cells *in vitro* and *in vivo*. Müller cells proliferate and produce pro-angiogenic factors such VEGF to promote proliferative DR. Moreover, the NLRP3 inhibitor MCC950 and the prodrug of epigallocatechin-3-gallate can reduce high glucose-induced upregulation of NLRP3 inflammasome-associated proteins and pro-angiogenic factors (Du et al., 2020). This finding illustrates the activation of the NLRP3 inflammasome pathway in Müller cells in DR and provides support for a specific role of NLRP3 in the neovascularization of PDR.

Microglia are resident immune cells that monitor retinal surroundings and remove metabolic waste in the retina (Rathnasamy et al., 2019). It occurs in various retinal disease states and migrates to the outer nuclear layer instead of spreading over the internal and outer plexiform layer, internal nuclear layer and ganglion cell layer in a normal state (Vecino et al., 2016; Rathnasamy et al., 2019). A recent study showed that S100 protein (S100A12) is a proinflammatory trigger in hyperglycemia-induced retinal microglial activation and inflammation by regulating NLRP3 activation. It also induces IL-1β and IL-18 release from microglia through a miR-30a-dependent mechanism (Dong and Wang, 2019). Another study confirmed that high glucose induces retinal microglial pyroptosis through NLPR3 inflammasome signaling. High-glucose stimulation decreases cell viability, enhances LDH release and upregulates the protein expression of IL-1β, NLRP3, cleaved caspase-1, and cleaved GSDMD. NLRP3 inhibitors or caspase-1 inhibitors can inhibit NLRP3 inflammasome activation and prevent retinal microglia from undergoing pyroptosis (Huang et al., 2021). Furthermore, retinal ischemia and reperfusion injury, as the basis of multiple retinal diseases, including DR, glaucoma and retinal artery occlusion, has been demonstrated to promote retinal microglial pyroptotic death, which is linked with lncRNA H19 (Hartsock et al., 2016). A recent study proved that NLRC5 promotes ganglion cell death in ischemic retinopathy through inducing microglial pyroptosis (Deng et al., 2021). In addition, high glucose increases IL-1β expression in rat retinal neural cells, which in turn impacts retinal microglial cell proliferation (Baptista et al., 2017). These studies suggest that activated retinal microglia-related inflammation and pyroptosis under high-glucose conditions may play a significant role in the development of DR.

Finally, although RPE cells are not traditionally included in the retinal neurovascular unit and are not viewed as key for the pathophysiology of DR, several studies have recently found that RPE pyroptosis is also involved in the development of DR and have tried to elucidate the mechanisms underlying RPE cell pyroptosis in DR. High glucose inhibits RPE cell proliferation and promotes RPE cell apoptosis and pyroptosis. Pyroptotic-associated proteins (Caspase-1, Gasdermin D, NLRP3, IL-1β and IL-18) are upregulated under high-glucose stimulation, which is alleviated by overexpressing METTL3 by targeting miR-25–3p and aggravated by knocking down METTL3 in RPE cells (Zha et al., 2020). Another study found that high glucose (50 mM) induced ROS-mediated pyroptotic cell death in ARPE-19 cells. Overexpression of miR-130a abrogated high glucose-induced ARPE-19 cell pyroptosis by targeting the TNF-α/SOD1/ROS axis (Xi et al., 2020). Recently, the STAT3 axis was reported to be involved in proptosis by elevating the transcription of the GSDMC gene and increasing STAT3 phosphorylation in DR rats. MiR-20b-5p rescues ARPE-19 cell pyroptosis by targeting STAT3 (Liang et al., 2021). The TXNIP-Trx-TrxR redox pathway may participate in RPE dysfunction in DR. Auranofin, as a thioredoxin reductase 1 (TrxR1) and TrxR2 inhibitor, can activate the NLRP3 inflammasome to release proinflammatory caspase-1 and cause pyroptotic cell death as measured by LDH leakage. NLRP3 and Caspase-1 inhibitors (NLRP3 and Caspase-1 inhibitors) can significantly reduce auranofin-induced LDH release and pyroptosis (Yumnamcha et al., 2019). Overall, the role of RPE pyroptosis in the mechanism of DR has gradually become a concern.

Furthermore, numerous studies have confirmed that NLRP3 inflammasome activation is associated with DR *in vivo*. Increased gene and protein expression of NLRP3, ASC, and caspase-1 in peripheral blood mononuclear cells of adults with DR compared with that in normal people. Meanwhile, elevated expression of NLRP3 and ASC was observed in fibrovascular membranes from proliferative DR patients (Chen et al., 2018). The expression levels of the inflammasomes NLRP3 and caspase-1 and the proinflammatory factors IL-1β and IL-18 were notably observed in vitreous of DR patients (Loukovaara et al., 2017). These studies suggest that the initiation of NLRP3 inflammasomes may promote the progression of DR. However, we need to be wary of the literature on inflammasome activation and pyroptosis in DR. As the critical role of gasdermin in pyroptosis was not established until 2015, earlier studies on pyroptosis were limited to confirmation of inflammasome and caspase-1 activation in DR (He et al., 2015). The N-terminal GSDMD domain can perforate the plasma membrane and form membrane pores, which play a crucial role in pyroptosis, further leading to cell swelling, cell content release and lytic cell death. Thus, an activated inflammasome, mature caspase-1, and the release of inflammatory cytokines in retinal cells do not necessarily mean that pyroptosis occurs in these cells. However, very few studies have directly demonstrated that the pyroptotic executor, the N-terminal GSDMD domain, is activated in DR. Future studies targeting GSDMD-N are needed to assess its value as a therapeutic target for DR.

### Overview of Necroptosis

**FIGURE 2:**
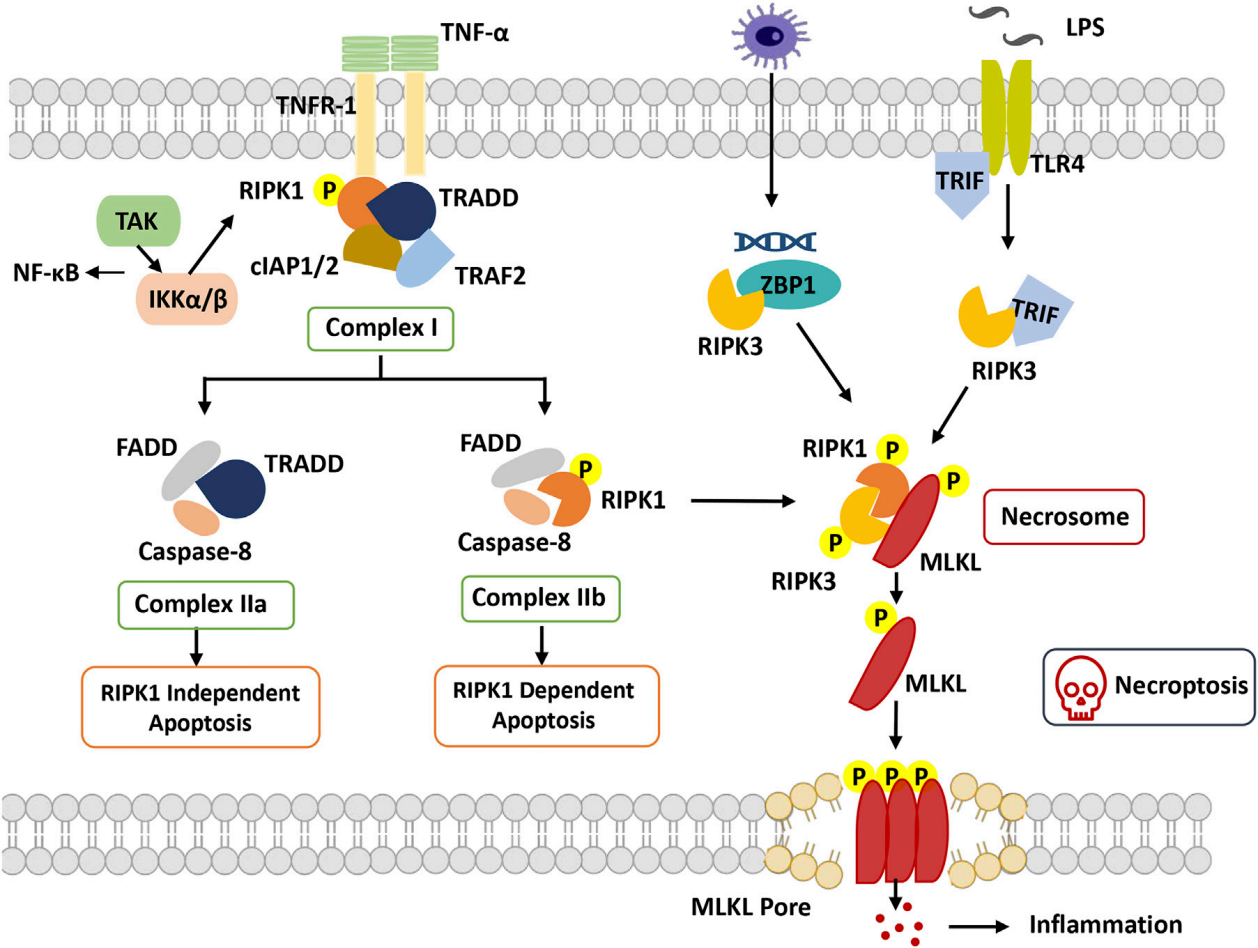
Overview of necroptosis. With stimulation of TNFα, TNFR1 recruits TRADD and binds with RIPK1, combining with TRAF2 and cIAP1/2 to form complex I. RIPK1 can be ubiquitinated by cIAP1/2, which promotes dissociation of TRADD and RIPK1 from complex I to trigger apoptosis or necroptosis. In apoptosis, FADD and caspase-8 are recruited to TRADD and RIPK1, respectively, to form complex IIa and complex IIb. Caspase-8 is activated, and apoptosis is induced. In necroptosis, when caspase-8 is inhibited, RIPK1 phosphorylates and activates RIPK3 to form necrosome complexes and phosphorylate MLKL. Phosphorylated MLKL undergoes oligomerization and migrates to the plasma membrane, where it induces necroptosis by initiating membrane rupture. Abbreviation: cIAP, cellular inhibitors of apoptosis protein; FADD, Fas-associated death domain; IKKα/β, IkB kinase α/β; MLKL, mixed lineage kinase domain-like protein; RIPK1, receptor-interacting protein kinase 1; RIPK3, receptor interacting protein kinase 3; TAK1, beta-activated kinase 1; ITLR, toll-like receptor; TRADD, TNF receptor-associated death domain; TRAF, TNF receptor-associated factor; TRIF, TIR-domain-containing adapter-inducing interferon-β; ZBP1, Z-DNA-binding protein 1.

Necroptosis is a caspase-independent RCD characterized by cell swelling, mitochondrial membrane permeabilization, and membrane rupture followed by the release of cellular contents and proinflammatory factors (Kaczmarek et al., 2013). It is executed in response to specific stimuli and is involved in the activation of special signal transduction pathways. Several necroptotic inducers comprise tumor necrosis factor α (TNFα), the CD95 receptor/Fas ligand complex, and other members of the TNF superfamily and small molecules causing serious cell stress, etoposide, and viral infections. Primary factors involved in necroptosis-associated cellular signal transduction include receptor interacting serine/threonine-protein kinase 1 (RIPK1), receptor interacting serine/threonine-protein kinase 3 (RIPK3), and mixed lineage kinase ligand (MLKL), which coordinate multiple protein complexes to conjointly execute necroptotic cell death (Grootjans et al., 2017) (Figure 2).

RIPK1 was the first protein found to be crucial for Fas-, TNF- and TNF-related apoptosis-inducing ligand (TRAIL)-induced necroptosis, including the N-terminal kinase domain, C-terminal death domain (DD), and an intermediate domain (Holler et al., 2000). Based on the posttranslational modifications of the intermediate domain and death domain, RIPK1 orchestrates different multiprotein complexes (Complexes I, IIb and IIc) (Festjens et al., 2007). The formation of necroptosis-specific protein complexes has been confirmed for TNF signal transduction. TNF receptor 1 (TNFR1) recruits TNF receptor type 1 associated death domain (TRADD), TNF receptor associated factor 2 (TRAF2), cellular inhibitors of apoptosis 1 and 2 (cIAP1 and cIAP2) and RIPK1 to form Complex I to induce RIPK1 polyubiquitination, in which the C-terminal DD of RIPK1 is responsible for the interaction with TNFR1 and TRADD (Festjens et al., 2007). The C-terminal DD participates in RIPK1 dimerization and activation during its transition from Complex I to Complex IIb(Meng et al., 2018). The N-terminal kinase domain is involved in S161 and S166 autophosphorylation and RIPK3 activation during the formation of Complex IIc. The intermediate domain is responsible for a scaffold for K63 and linear M1 ubiquitylation, which recruits the TGFβ activated kinase 1 (TAK1) complex and the IκB kinase (IKK) complex. It is also required for the recruitment of RIPK3 and for binding to other RHIM-containing proteins (Vandenabeele et al., 2010).

RIPK3 is homologous to RIPK1 and consists of a similar N-terminal kinase domain, an intermediate domain, and an RHIM domain at its C-terminus but lacks the C-terminal death domain compared to RIPK1(Wu et al., 2014). Through the RHIM-domain interaction, RIPK3 and RIPK1 ultimately assemble Complex IIc, which in turn causes RIPK3 autophosphorylation. Subsequently, RIPK3-mediated phosphorylation activates MLKL attracted by the complex, and then the RIPK3-MLKL complex transfers into the cytosol to construct Complex IIc(Weber et al., 2018).

Another crucial mediator of necroptosis, MLKL, is the sole and main effector of necroptosis. Its N-terminal four-helical bundle domain can directly or indirectly cause membrane pore formation (Hildebrand et al., 2014). MLKL interacts with RIPK3 through its kinase domains, in which phosphorylation of RIPK3 at Ser277 is necessary for their interaction. Activated MLKL forms amyloid-like oligomers and is transferred to the cytoplasmic membrane, facilitating the terminal steps of necroptosis (Weber et al., 2018).

TNF-induced necroptosis is the canonical necroptotic pathway. Upon TNF stimulation, TNF receptor 1 recruits multiple proteins (TRADD, TRAF2, cIAP1/2 and RIPK1) to assemble TNFR1 Complex I. The key step of necroptosome (Complex IIc) formation is RIPK1 deubiquitylation and caspase 8 inhibition, as well as the kinase activities of RIPK1 and RIPK3. RIPK3 phosphorylates phosphoglycerate mutase family member 5 (PGAM5) to activate dynamin-1-like protein (DRP1), which causes mitochondrial fission and participates in the necroptosis process. Moreover, MLKL phosphorylation is performed by RIPK3 followed by the formation of MLKL oligomers that transfer to the membrane as MLKL ion channels, leading to necroptosis (Khan et al., 2021).

### Necroptosis and Diabetic Retinopathy

Previous studies have implicated necrosis in the development of DR, such as increased necrotic cell death of pericytes in the retinas of diabetic rats (Feenstra et al., 2013). Subsequent studies have found that necroptosis plays a key role in the pathogenesis of blindness diseases, including glaucoma, age-related macular degeneration and retinitis pigmentosa, which are involved in the death of RGCs, RPE cells and cone cells, respectively (Peng et al., 2020). Similarly, it has been extensively reported that necroptosis contributes to neuronal damage in a retinal ischemia–reperfusion injury model through the ERK1/2-RIP3 pathway, which can be inhibited by necrostatin 1 *in vitro* and *in vivo* experiments (Dvoriantchikova et al., 2014; Gao et al., 2014). Retinal ischemia is involved in various disorders, including DR, glaucoma, optic neuropathies and retinopathy of prematurity. Death-associated protein (Daxx), a novel substrate of RIP3, has been implicated in ischemic necrosis of retinal cells. The phosphorylation of Daxx by RIP3 plays a crucial role in ischemic necrosis in rat RGCs(Lee et al., 2013). However, only a few studies reported retinal cell necroptotic death in DR. Our previous study indicated that high glucose could induce the expression of RIPK1/RIPK3 and phosphorylated RIPK1/RIPK3, and necrostatin-1 could effectively protect RGCs from glucose-induced necroptosis (Gao et al., 2021). Blue light acts on the mitochondria of RGCs and has the potential to elicit necroptosis through RIP1/RIP3 to stimulate RGC death in diseases such as glaucoma and DR, a process that is dependent on mitochondria (Del Olmo-Aguado et al., 2016). Studies associated with necroptosis in DR are mainly focused on RGC cells, and whether other retinal cells undergo necroptosis under high glucose induction still needs further investigation. Further clarification of the definition of necroptosis and the pathways involved may be necessary to better understand and identify this process in DR.

### Overview of Ferroptosis

**FIGURE 3:**
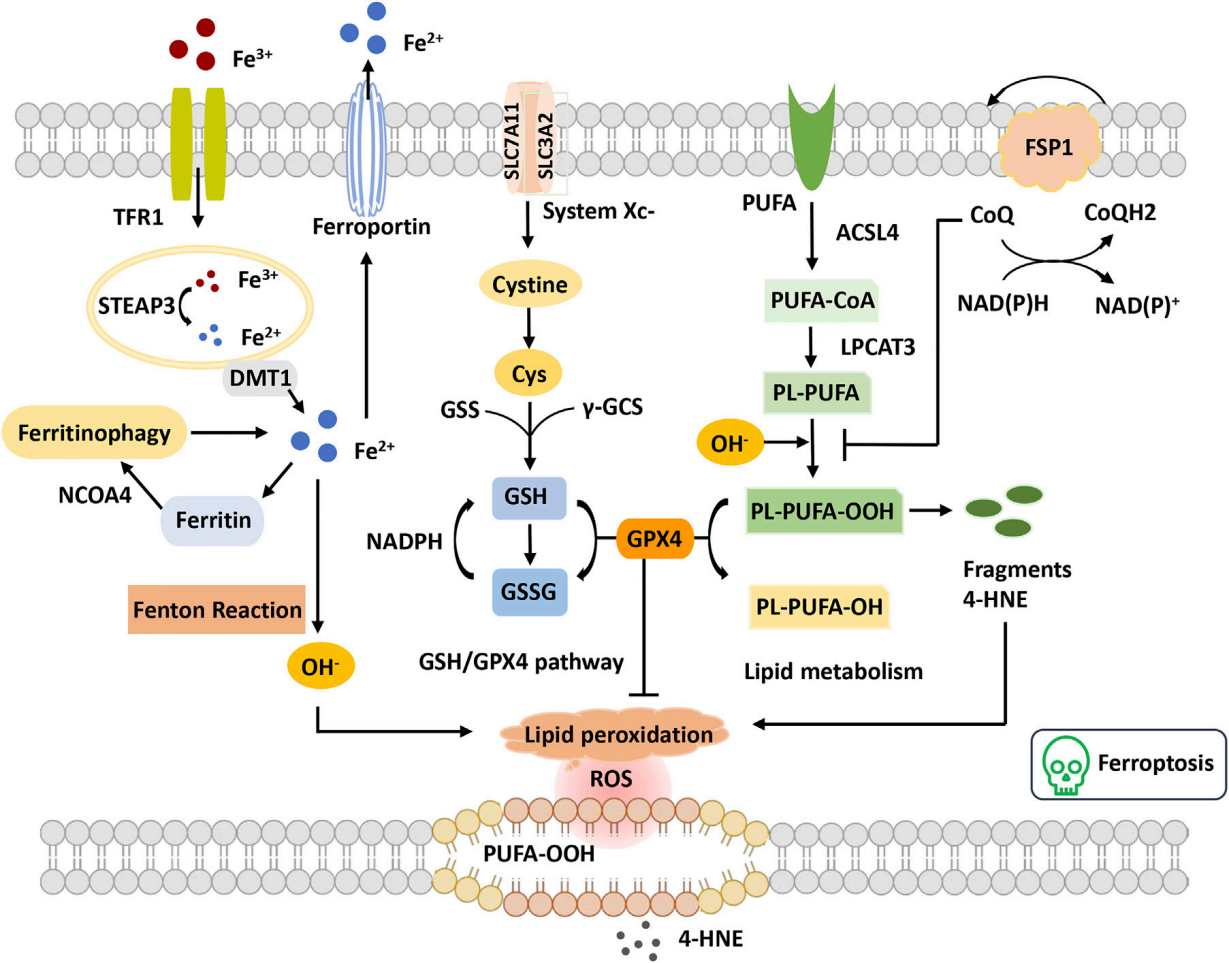
Overview of ferroptosis. The figure shows four systems regulating ferroptosis. Iron metabolism, Fe^3+^ is transferred into the cell through TFR1 and reduced to Fe^2+^ by STEAP3. Fe^2+^ is transported to the cytoplasm through DMT1. Excess Fe^2+^ causes the Fenton reaction and the initiation of lipid peroxidation, OH^−^. GSH-GPX4 pathway GSH synthetization is catalyzed by GSS and γ-GCS. GPX4 converts GSH to GSSG to inhibit lipid ROS production. Lipid metabolism and OH^−^ promote the oxidation of PUFAs on the cell membrane, leading to the accumulation of PE-PUFA-OOH and 4-HNE generation. PE-PUFA-OOH can be repaired by GPX4 to reduce lipid hydrogen. In the mevalonate pathway, FSP1 is recruited to the cell membrane and decreases CoQ and suppresses lipid peroxidation, which mediates a nonmitochondrial CoQ antioxidant system. Abbreviations: ACSL4, acyl-CoA synthetase long-chain family member 4; CoQ, coenzyme Q; Cys, cysteine; DMT1, divalent metal transporter 1; FSP1, ferroptosis suppressor protein 1; GPX4, glutathione peroxidase 4; GSH, glutathione; GSS, glutathione synthetase; GSSG, oxidized glutathione; LPCAT3, lysophosphatidylcholine acyltransferase 3; NCOA4, nuclear receptor coactivator 4; PUFA, polyunsaturated fatty acid; PUFA-CoA, polyunsaturated fatty acyl CoA; PLOOH, phospholipid hydroperoxides; ROS, reactive oxygen species; STEAP3, the six-transmembrane epithelial antigen of the prostate 3; TFR, transferrin receptor; γGSC, γ-glutamylcysteinyl synthetase; 4-HNE, 4-hydroxynonenal.

Ferroptosis is defined as an iron-catalyzed form of regulatory cell death that occurs through the excessive peroxidation of polyunsaturated fatty acids (PUFAs) (Dixon et al., 2012) (Figure 3). Since the proposal of the concept of ferroptosis, ferroptosis has attracted much interest. Morphologically, cells suffering from ferroptosis usually show necrosis-like morphological changes, including cell swelling, plasma membrane integrity disruption and swelling of cytoplasmic organelles, along with abnormal mitochondria with reduced or absent cristae, a condensed membrane, and a ruptured outer membrane at the ultrastructural level (Dixon et al., 2012; Friedmann Angeli et al., 2014). Furthermore, ferroptotic cells do not present features of apoptosis, and knockdown of necroptotic key mediators cannot prevent ferroptosis (Friedmann Angeli et al., 2014). Ferroptosis is an ROS-dependent cell death involved in two core biochemical features, namely, iron accumulation and lipid peroxidation. Ferroptosis execution relies on an iron-catalyzed excessive peroxidation of PUFA-containing phospholipids in cell membranes (Yang et al., 2016). The classical ferroptosis activator erastin inhibits the antioxidant system to accumulate the intracellular iron concentration. Excessive iron can directly create excess ROS *via* the Fenton reaction to cause oxidative damage and then activate lipoxygenase (LOX) or prolyl hydroxylases to disturb lipid peroxidation and oxygen homeostasis (Chen X. et al., 2020). Cells maintain relatively stable iron metabolism by regulating intracellular iron uptake, utilization, storage, and export. Disorganized iron metabolic processes can promote or inhibit ferroptosis, depending on the levels of intracellular free iron (Chen X. et al., 2020). Nonenzymatic lipid peroxidation is a free radical-driven chain reaction in which ROS trigger the excessive peroxidation of PUFAs(Ayala et al., 2014). Enzymatic lipid peroxidation is mediated in a regulated manner by the LOX family that can catalyze the dioxygenation of PUFAs to generate various lipid hydroperoxides. However, the clear mechanism of lipid peroxidation leading to ferroptosis remains elusive. It is hypothesized that it might be associated with the formation of a structured lipid pore, membrane fluidity and structure modification, increased membrane permeability, loss of membrane integrity, or destabilized membrane by excessive oxidant access, causing pore and micelle formation (Agmon et al., 2018; Cheng et al., 2018). In addition, it has been reported that the toxicity of lipid peroxidation might inactivate proteins involved in essential cellular processes to promote ferroptosis (Zhong and Yin, 2015).

In recent years, the mechanistic understanding of ferroptosis has made rapid progress. Two important cellular components have been identified, the inhibition of which can induce cell ferroptosis: glutathione peroxidase 4 (GPX4) and system x_c_^−^ (cystine/glutamate antiporter), inhibited by the compounds RSL3 and erastin, respectively (Dixon et al., 2014; Yang et al., 2014). The role of the cysteine-glutathione (GSH)- GPX4 mechanism in inhibiting ferroptosis has been identified, in which phospholipid hydroperoxide (PLOOH) acts as a ferroptosis executioner. Canonical ferroptosis is induced through the GPX4-regulated ferroptotic pathway. GPX4 is the primary enzyme that catalyzes the reduction of PLOOH and reduces phospholipids and cholesterol hydroperoxides to their corresponding alcohols (Maiorino et al., 2018). The inactivation of GPX4 deprives cells of cysteine, an essential cellular antioxidant and a building block of GSH, which leads to PLOOH accumulation and consequently rapid and unrepairable damage to the plasma membrane, causing ferroptosis (Jiang et al., 2021). Furthermore, unrestrained lipid peroxidation is the hallmark of ferroptosis. Initiating lipid peroxidation requires the extraction of diallyl hydrogen atoms between the two carbon-carbon double bonds from the polyunsaturated fatty acyl part of the phospholipid (PUFA-PL) bound to the lipid double layer to form the carbon center radical, which then reacts with molecular oxygen to produce peroxide radicals. Without conversion to lipid hydroperoxides and reduction to the corresponding alcohols, propagation of the radical-mediated reaction leads to the formation of numerous secondary products that disrupt membrane integrity and ultimately rupture organelles and membranes (Jiang et al., 2021). Additionally, multiple lines of evidence have demonstrated that metabolism plays a crucial role in ferroptosis. Various metabolic events (including lipogenesis, autophagy, and the mitochondrial TCA cycle) and signaling pathways (including the E-cadherin-NF2-Hippo-YAP pathway, glucose-regulated AMPK signaling, and p53 and BAP1 tumor suppressor functions) are involved in the regulation of ferroptosis (Gao et al., 2015; Gao et al., 2016; Jiang et al., 2021). For example, cysteine deprivation-induced ferroptosis requires glutamine metabolism or glutaminolysis, which directly links ferroptosis to cellular metabolism (Gao et al., 2015).

### Ferroptosis and Diabetic Retinopathy

Although the specific physiological function of ferroptosis has not been clearly demonstrated, the role of ferroptosis in human diseases has been established. Recent studies have reported a significant role of ferroptosis in the progression of diabetes mellitus and its multiple complications, including diabetic kidney disease, diabetic neuropathy, diabetic osteoporosis and DR (Yang and Yang, 2022). One recent study found that extracellular glia maturation factor-β (GMFB), a neurodegenerative factor upregulated in the vitreous at a very early stage of diabetes, can translocate the ATPase ATP6V1A from the lysosome and damage lysosomal acidification in RPE cells, which causes ACSL4 protein accumulation and catalyzes the production of lethal lipid species and induces ferroptosis in RPE cells, ultimately impairing the function of the retina. In a diabetic rat model, the ferroptosis inhibitor liproxstatin-1 (LX-1), GMFB antibody, lysosome activator NKH477, and CMA activator QX77 all present strong protective effects on retinal function (Liu et al., 2022). An *in vitro* study in ARPE-19 cells showed that circ-PSNE1 was increased in high glucose-treated ARPE-19 cells. Knockdown of circ-PSEN1 regulates intracellular concentrations of GSH, malondialdehyde, and ferrous iron, increases the cell survival rate and suppresses ferroptosis in high glucose-treated ARPE19 cells. Circ-PSEN1 mitigates high glucose-induced ferroptosis via the miR-200b-3p/cofilin-2 axis (Zhu et al., 2021). Similarly, the expression of miR-138–5p is upregulated in RPE cells treated with high glucose, which reduces Sirt1/Nrf2 activity and antioxidant expression and ultimately induces RPE cell ferroptosis. Astragaloside-IV alleviates high glucose-induced ferroptosis in RPE cells by disrupting the expression of miR-138–5p/Sirt1/Nrf2(Tang et al., 2022). In addition, a novel miR-338–3p/SLC1A5 axis has been reported that regulates oxidative stress-mediated RPE cell ferroptosis to modulate DR progression. High glucose upregulates miR-338–3p to cause SLC1A5 degradation in RPE cells, leading to oxidative stress-mediated ferroptosis. Thus, targeting the miR-338–3p/SLC1A5 axis is effective in increasing the resistance of the RPE to high glucose-induced ferroptosis (Zhou et al., 2022).

In addition to RPE cell changes, retinal capillary endothelial cell (RCEC) dysfunction also contributes to diabetic retinal damage early in DR. High glucose suppresses human RCEC growth and induces ferroptosis, which is reversed by a ferroptotic inhibitor. RCECs treated with high glucose exhibited increased TRIM46 expression, and TRIM46 overexpression decreased cell resistance against high glucose-induced ferroptosis. TRIM46 interacted with GPX4 to promote its ubiquitination. Overexpression of GPX4 ameliorates the effects of TRIM46 upregulation and protects RCECs against ferroptosis. Thus, TRIM46 contributes to high glucose-induced ferroptosis in human RCECs by facilitating GPX4 ubiquitination (Zhang et al., 2021). These studies indicate that ferroptosis plays a crucial role in damage to RPE cells and RCECs in DR progression. In addition, Fe^2+^ intravitreal injection increases the levels of oxidative stress markers and components of ferroptosis in the retina and induces photoreceptor death dependent on ferroptosis (Shu et al., 2020). However, whether ferroptosis participates in hyperglycemia-induced retinal neuron, pericyte or glial cell loss dependent on ferroptosis is still unclear and requires further exploration.

## Novel Regulated Cell Death as Therapeutic Targets for Diabetic Retinopathy

Based on the evidence above, novel RCD may contribute to retinal cell death and the pathogenesis of DR progression. Different cell death modes may all be candidates for therapeutic targets of DR.

Pyroptosis occurrence has been confirmed in high glucose-induced retinal cells and DR rat models. Some studies have reported that several pyroptosis or inflammasome inhibitors have been tested in DR models *in vivo* and *in vitro*. MCC950, as the most potent and specific NLRP3 inhibitor, has been confirmed to attenuate NLRP3 inflammasome initiation and IL-1β secretion and rescue cell death in high-glucose-stimulated HRECS *via* the NLRP3–NEK7 pathway (Zhang et al., 2017). Epigallocatechin-3-gallate (EGCG), the key bioactive compound found in tea, can inhibit Müller cell proliferation and proangiogenic factor production by suppressing the ROS/TXNIP/NLRP3 inflammasome axis in streptozotocin (STZ)-stimulated DR mouse models (Du et al., 2020). The connexin-32 hemichannel inhibitor can inhibit inflammation of the retina in DR by preventing NLRP3 inflammasome complex assembly, Müller cell activation, and the release of proinflammatory cytokines (Louie et al., 2021). Another study suggested that connexin 43 hemichannels may mediate RPE disruption that occurs in DME in an ATP release/inflammasome pathway activation-dependent manner, which can be blocked by peptide 5 (Kuo et al., 2020). Moreover, high glucose-stimulated P2X7 purinergic receptor (P2X7R) mediates NLRP3 inflammasome activation in DR pathogenesis. H3 relaxin treatment can remarkably attenuate P2X7R and NLRP3 inflammasome activation in HRMEC, which further decreased pyroptosis and migration of HRMECs in response to AGEs mediated by P2X7R activation of the NLRP3 inflammasome (Yang et al., 2020). Several natural products can also be potential pharmacological drugs for DR by suppressing inflammasome-mediated cell death. Palbinone has been reported to exhibit protective effects against DR through the downregulation of IL-18 and IL-1β levels, enhancement of antioxidant capacities, and negative regulation of inflammasome component expression (NLRP3, cleaved caspase-1, IL-1β and ASC) by activating the Nrf2 signaling pathway (Shi et al., 2020). Similarly, sulforaphane, an isothiocyanate found in cruciferous vegetables, stimulates the Nrf2 pathway and remarkably attenuates the retinal expression levels of NLRP3, cleaved-caspase 1, ASC, and cleaved IL-1β in STZ-induced mouse models (Li et al., 2019). Gambogic acid, as the active component of gambode resin, inhibits TXNIP/NLRP3 activation by downregulating TXNIP, NLRP3, ASC, cleaved-caspase 1, and cleaved-IL 1β levels to flatten the inflammatory response in RPE cells by modulating the Nrf2 signaling pathway (Chen et al., 2021). In addition, methylene blue, as an oxidation–reduction molecule, can significantly decrease NLRP3 inflammasome activation and attenuate IL-1β and IL-18 secretion in STZ-stimulated diabetic rat models (Hao et al., 2019). Hydrogen sulfide (H_2_S) has been shown to protect against oxidative stress injury and inflammation in high glucose-induced RPE cells by inhibiting ROS formation and NLRP3 inflammasome activation (Wang et al., 2019). Resolvin D1 treatment efficiently decreased IL-1β and IL-18 secretion by suppressing the NF-kB pathway in the retinal tissue of DR rats (Yin et al., 2017). These findings suggest that pyroptosis-related proteins and inflammasomes may be potential novel therapeutic targets for DR.

Targeting necroptosis might be an alternative method to reverse cell death in the development of DR. Necroptosis contributes to neuronal damage in the retinal ischemia–reperfusion injury model. Necroptosis inhibitors (necrostatin-1) exemplified the treatment effect on RGC death induced by high glucose (Gao et al., 2021). However, due to the few studies and limited current therapeutic options, it remains necessary to further evaluate necroptosis as a potential novel therapeutic target for DR.

With the deeper recognition of the ferroptosis mechanism, several specific inhibitors of ferroptosis have been discovered, such as ferrostatin-1, liproxstatin-1 and deferoxamine, which have been studied in diabetes and its complications. ACSL4, as the substrate of chaperone-mediated autophagy, is an essential component of ferroptosis. It has been reported that GMFB induces ferroptosis by impairing chaperone-mediated autophagic degradation of ACSL4 in early DR. The ferroptosis inhibitor liproxstatin-1 is effective in a diabetic rat model to prevent early DR and maintain normal visual function (Liu et al., 2022). Some natural products have been developed to suppress cell ferroptosis. Quercetin, resveratrol, germacrone and cryptochlorogenic acid all have been reported to have potential anti-ferroptosis properties in diabetes mellitus or its complications (Yang and Yang, 2022). In a DR *in vitro* model, astragaloside-IV, a high-purity natural product extracted from *Astragalus* membranaceus, alleviates high glucose-induced ferroptosis in RPE cells by disrupting the expression of miR-138–5p/Sirt1/Nrf2(Tang et al., 2022). In addition, downregulation of TRIM46 increases RCEC resistance against high glucose-induced perroptosis. Administration of RSL3, a ferroptosis agonist, was able to reverse the protective effects of TRIM46 silencing (Zhang et al., 2021). These studies suggest that ferroptosis is a promising therapeutic target for DR. Further studies are needed to identify novel targets of ferroptosis for the development of effective drugs for DR treatment.

## Conclusion

In conclusion, we summarize the role and possible mechanisms of RCD in DR, including pyroptosis, necroptosis and ferroptosis. Cell death seems to be a remarkable feature in the progression of DR, and several retinal cell types have been demonstrated to suffer different forms of RCD. PANoptosis and cuproptosis are concentrated on the role of protection against infection and anti-tumor therapy currently. The field of these 2 cell death pathways is nascent in many ways, thus further studies are needed to explore the association with DR. Moreover, further studies are needed to reveal how different forms of RCD interact under retinal hyperglycemia conditions and what key factors ultimately determine the specific death type to which retinal cells will be attributed in a pathological state. This is also crucial for the development of therapeutics targeting RCD. Finding potential links between RCD facilitates a deeper understanding of the interacting mechanisms of cell death in DR. The microenvironment around retinal cells in a hyperglycemic state also deserves attention from researchers later, which may partly determine the type of cell death. Based on the evidence from different retinal cell death in DR in *in vitro* and animal model studies, there is reason to assume that novel RCD may act as a therapeutic target for DR. Drugs that target ferroptosis may provide new treatment strategies for patients with diabetes. Targeting pyroptosis-mediated cell death in DR has attracted widespread attention and may be a promising strategy. In the future, targeted drugs can be designed to block these RCD pathways in DR to achieve effective treatment and improve prognosis.
